# Mechanisms of Serrated Flow and Microstructural Evolution in MarBN Steel

**DOI:** 10.3390/ma16196411

**Published:** 2023-09-26

**Authors:** Tongfei Zou, Quanyi Wang, Yubing Pei, Ting Mei, Hong Zhang

**Affiliations:** 1 Failure Mechanics and Engineering Disaster Prevention and Mitigation Key Laboratory of Sichuan Province, College of Architecture and Environment, Sichuan University, Chengdu 610065, China; ephrysz@outlook.com (T.Z.); scuwangquanyi@foxmail.com (Q.W.); 2 Key Laboratory of Deep Underground Science and Engineering, Ministry of Education, Sichuan University, Chengdu 610065, China; 3 State Key Laboratory of Long-Life High Temperature Materials, Dongfang Turbine Co., Ltd., Deyang 618000, China; peiyubing@dongfang.com; 4 AVIC Guizhou Honglin Aerodynamic Control Technology Co., Ltd., Guiyang 550000, China; meiting0725@sina.com

**Keywords:** MarBN steel, tensile behavior, serrated flow, microstructure

## Abstract

The mechanisms of serrated flow and microstructural evolution in MarBN steel were studied under two strain rates (5 × 10^−3^ s^−1^ and 5 × 10^−5^ s^−1^) at room temperature and high temperatures (430 °C and 630 °C). The experimental results show that the type-C serrations occurred at all temperatures under a high strain rate of 5 × 10^−3^ s^−1^. In contrast, type-B serrations occurred at 430 °C and 630 °C under a low strain rate of 5 × 10^−3^ s^−1^, indicating that the type of serrated flow was related to the strain rate. The microstructural results reveal that pinning and unpinning dislocation under both strain rates were responsible for the serrations at both strain rates.

## 1. Introduction

Serrated flow [1], or the Portevin-Le Chatelier (PLC) effect [2], viewed in the stress–strain curve during plastic deformation is a plastic instability behavior that has been studied in various kinds of aluminum alloys [3,4], nickel-based superalloys [5,6,7], magnesium alloys [8,9,10], and steel [11,12,13,14]. The primary characteristic of the PLC effect is a serrated oscillation pattern in tensile curves after achieving the yield point resulting from repetitive nucleation and the development of localized deformation regions [15,16]. The dynamic-strain-aging (DSA) interaction between mobile dislocations and solute atom clouds during plastic deformation has been widely accepted as an explanation for the serrations of the stress–strain curve during static tensile tests. Subsequently, a critical strain mechanism [17] was proposed to define the ability to unpin dislocations from solute atoms when a serrated flow occurs, where the first serrated flow that appears in a plastic flow can be defined as critical strain. S.-K. Oh et al. [15] investigated the variation of A-type serrations with strain rate in a plastic flow using Fe-18Mn-0.55 C steel. J. Brechtl et al. [18] undertook specific modelling and analysis of serrated flow behavior using the refined composite multiscale entropy method on an experimental basis.

MarBN steel, strengthened by boron and MX nitrides, is one of the advanced high-strength alloys used in the rotating components of the turbine engine, and it is expected to be one of the candidate materials for A-USC power plants [19]. Zhang et al. [20] studied the low-cycle fatigue (LCF) behavior of MarBN steel at room temperature (RT) and high temperatures, indicating that cyclic softening is related to the size of laths and dynamic recrystallization and grain rotation at RT and high temperatures, respectively. They also investigated the tension–compression asymmetry during the LCF of MarBN steel under different loading modes at RT [21]. However, it is essential to better understand the corresponding mechanical properties and address the plastic instabilities over the entire range of service temperatures. Therefore, the purpose of the present paper is to investigate the mechanism of serrations in MarBN steel under strain rates of 5 × 10^−3^ s^−1^ and 5 × 10^−5^ s^−1^ at RT, 430 °C, and 630 °C based on a tensile experiment and transmission electron microscopy (TEM) characterization.

## 2. Experimental Procedure

MarBN steel was employed in the present work, and its composition (in wt. %) is as follows: 0.10 C, 9.16 Cr, 0.06 Si, 0.20 Mn, 0.20 Mo, 0.40 Ni, 0.08 Nb, 2.95 W, 2.82 Co, 0.20 V, and Fe as balance. The more-detailed as-received microstructures of MarBN steel can be found in our previous reports [20,22,23]. Cylindrical tensile samples with a gauge diameter of 5 mm [20] were used, and uniaxial tensile tests were carried out on a SHIMADZU AGX 100 tensile tester equipped with a heating furnace, which can constrain temperature fluctuations to about ±3 °C. Tensile strain rates of 5 × 10^−3^ s^−1^ and 5 × 10^−5^ s^−1^ were employed at both RT and high temperatures (430 °C and 630 °C), and the tests were repeated three times for each set of experimental conditions to exclude potential errors. The specimens were heated to a specific temperature and held for 20 min to equalize the temperature distribution in the samples when performing elevated-temperature tensile tests. An extensometer with a 25 mm gauge length was used to monitor strain at all temperatures. After the completion of the tensile tests, metallographic examinations were performed, using TEM to analyze the microstructural patterns. TEM foils were prepared using a Struers Tenupol-5 double-jet thinning apparatus, utilizing liquid nitrogen for refrigeration to maintain the temperature at −25 °C. The voltage was 20 V, and the liquid environment was 90% anhydrous ethanol + 10% perchloric acid. Microstructural observations were performed on a field emission TEM (model FEI Tecnai G2 F20).

## 3. Results

**Figure 1:**
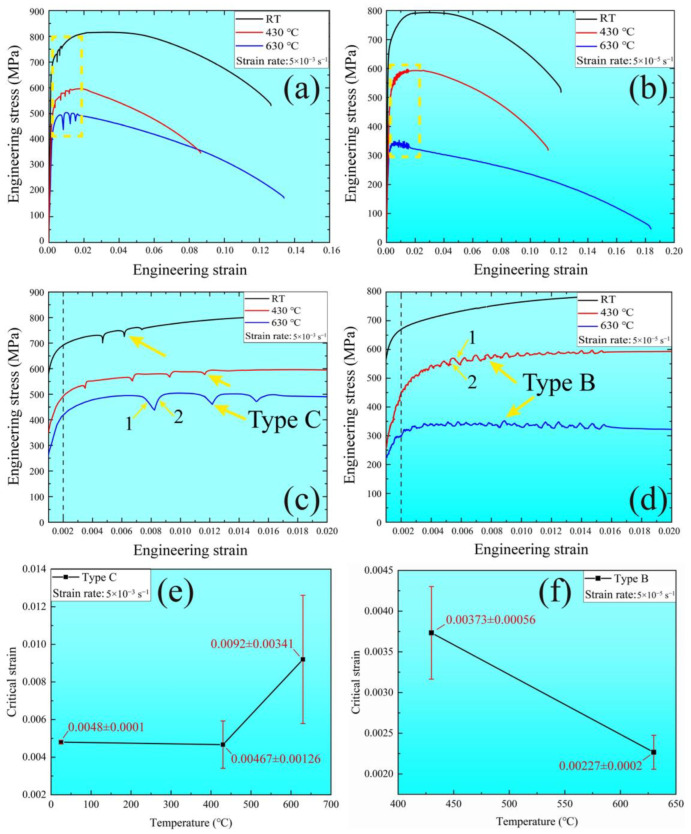
Stress–strain curves of tensile samples under two strain rates (5 × 10^−3^ s^−1^ and 5 × 10^−5^ s^−1^) at RT, 430 °C, and 630 °C: (**a**,**b**) stress–strain curves at strain rates of 5 × 10^−3^ s^−1^ and 5 × 10^−5^ s^−1^; (**c**,**d**) magnified sections of stress–strain curves indicated by yellow rectangle in (**a**,**b**), where arrows 1 and 2 represent the processes of dislocation unpinning and pinning, respectively; (**e**,**f**) temperature dependence of the critical strain for serrations of types C and B.

The stress–strain curves for MarBN steel at two strain rates (5 × 10^−3^ s^−1^ and 5 × 10^−5^ s^−1^) and temperatures ranging from RT to 630 °C (RT, 430 °C, and 630 °C) are presented in Figure 1a,b. Local magnified views of the stress–strain curves indicated by a yellow rectangle are addressed in Figure 1c,d, respectively, in which the distinct shapes of serrations are visible. At a strain rate of 5 × 10^−3^ s^−1^ under all three temperatures, type-C serrations, featuring abrupt loading drops [10,24], appeared after yielding. In contrast, when decreasing the strain rates, type-B serrations, characterized by oscillations around the stress–strain curve [9,25], appeared at a strain rate of 5 × 10^−5^ s^−1^ at 430 °C and 630 °C. Unpinning and pinning for dislocations via solute atoms [26] are indicated by numbers 1 and 2 in Figure 1c,d, respectively.

The temperature dependence of the critical strain for serrations of types C and B are shown in Figure 1e,f. The critical strain error bars for the three specimens under each set of experimental conditions are given in the diagram. At a strain rate of 5 × 10^−3^ s^−1^ (Figure 1e), the critical strain [27] decreases with an increase in the temperature and reaches a minimum at 430 °C. After that, it increases when increasing the temperature from 430 °C to 630 °C. The descending trend at low temperatures can be defined as normal behavior, while the ascending trend at high temperatures corresponds to an inverse behavior [28]. By contrast, at a strain rate of 5 × 10^−5^ s^−1^ (Figure 1f), the critical strain continuously decreases with the temperature increase from 430 °C to 630 °C. According to the previous research [14,29], the crucial factor for critical strain is the solute diffusion at low temperatures and the dislocation pinning strength at high temperatures. Therefore, these results indicate that the serrations in MarBN steel depend on the corresponding strain rates and temperatures.

## 4. Discussion

According to the PLC theory, the serrations occurring under various temperatures and strain rates can be ascribed to the interaction between dislocations and solute atoms [1,3,30], whereas DSA results from pinning dislocations induced by solute atoms, leading to the appearance of serrated flow. In essence, serration behavior is controlled by the pinning and unpinning of dislocations during plastic deformation. Therefore, a serrated flow is related to critical strain, which is dependent on the strain rate and temperature [17,31], as shown in Figure 1.

At a high strain rate of 5 × 10^−3^ s^−1^(Figure 1a,c,e), type-C serration occurred at all temperatures, indicating that the unpinning dislocation behavior appears at the beginning of plastic deformation due to the high vacancy concentration and dislocation density caused by the high strain rate [32]. This result agrees with that from a previous report for another alloy [9]. Therefore, the critical strain can be defined as the first unpinning of the pinning dislocation. The dislocation velocity (*V*) is proportional to the applied stress (*σ*) [33], such as V∝σ. Solute atoms can follow the dislocation during the pinning process, indicating the same velocity. The relative rate between them is zero. Therefore, the stress–strain curve is smooth, resulting from the lower applied stress compared to the critical stress, i.e., $\sigma <\sigma_{up}$. When tensile deformation continues, the external stress increases and becomes critical stress, and the dislocations begin to disengage from the solute atom cloud. Then, the relative velocity is greater than zero, resulting in unpinning dislocation. According to the theory of dislocations [34], the applied force ($F_{S}$) and velocity ($v_{s}$) of solute atoms following dislocations are related to temperature, such as $F_{S}\propto T^{2}$ and $v_{s}\propto \;T$, indicating that the critical stress of the unpinning process increases with an increase in temperature. Furthermore, at low temperatures, the critical strain and temperature are inversely proportional [28], defined as $\varepsilon_{c}\propto \;exp\;(-Q/kT)$, where *Q* is the activation energy for the movement of the solute atom cloud in the matrix and *k* is the Boltzmann constant. Therefore, the critical strain decreases with the increase in the temperature, as shown in Figure 1e at a low temperature, increasing the diffusibility of the solute atoms. In contrast, at high temperatures, the critical strain and temperatures are proportional [28], defined as $\varepsilon_{c}\propto \;T$, indicating that the critical strain increases with an increase in temperature.

By contrast, at a low strain of 5 × 10^−5^ s^−1^ (Figure 1b,d,f), the type-B serration occurred at all temperatures, which indicates that the pinning dislocation behavior appears during plastic deformation. This result agrees with that from a previous report on other alloys [7,9,14,35]. Critical strain can be defined as the first pinning mobile dislocation. According to Orowan’s rule [36], the strain rate is proportional to the dislocation density and velocity, i.e., $\dot{\varepsilon }\propto \;\rho v$, where $\dot{\varepsilon }$ is the strain rate, ρ is the dislocation density, and v is the dislocation velocity. At RT, the dislocation velocity is too low to pin a solute atom cloud because the strain is less than the critical strain for pinning dislocation, i.e., $\varepsilon \;<\;\varepsilon_{c}$. When the temperature increases, the dislocation density decreases under the constant strain rate of 5 × 10^−5^ s^−1^ [22,37,38], while the dislocation velocity increases. Therefore, the strain at high temperatures meets the critical strain for pinning dislocation, i.e., $\varepsilon \;\geq \;\varepsilon_{c}$. Additionally, the dislocation velocity increases with the increase in temperature, indicating that the pinning dislocations process increases with an increasing temperature. In other words, the critical strain for pinning dislocation decreases with an increase in temperature, as shown in Figure 1f.

**Figure 2:**
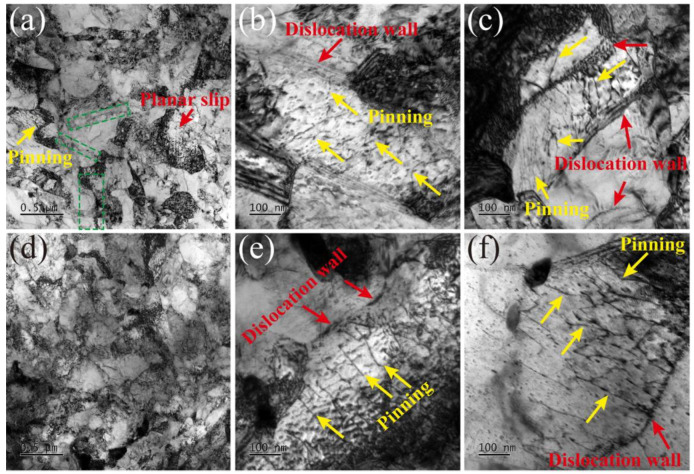
TEM images of microstructural evolution at (**a**) RT, where the green rectangular frames identifies the dynamic recrystallization behavior, (**b**) 430 °C, and (**c**) 630 °C with a strain rate of 5 × 10^−3^ s^−1^ and at (**d**) RT, (**e**) 430 °C, and (**f**) 630 °C with a strain rate of 5 × 10^−5^ s^−1^.

The results regarding microstructural evolution under various temperatures with strain rates of 5 × 10^−3^ s^−1^ and 5 × 10^−5^ s^−1^ after tensile failure are presented in Figure 2. At RT (Figure 2a), the dislocations are tangled along the grain boundaries due to the high strain rate, and a local planar slip (indicated by the red arrow) caused by the DSA can be observed [31]. Also, the dynamic recrystallization behavior, responsible for the serrated flow, is indicated by the green rectangle [7]. Limited pinning dislocation can also be observed within the grain. When the temperature reaches 430 °C and 630 °C (Figure 2b,c), dislocation walls (DWs) appear (indicated by the red arrow) due to the temperature. Several pinning and unpinning processes of dislocations were observed between different DWs. Therefore, the microstructural patterns can better prove the dislocation pinning and unpinning processes occurring during plastic deformation at a high strain rate of 5 × 10^−3^ s^−1^. In contrast, at RT with a strain rate of 5 × 10^−5^ s^−1^(Figure 2d), the pinning dislocation and planar slip are not observed, resulting in a smooth plastic curve. Compared to the strain rate of 5 × 10^−3^ s^−1^ at 430 °C and 630 °C, DWs and pinning dislocations (indicated in Figure 2e,f) correspond to the serrated flow under a specific strain rate. However, the dislocation density at 5 × 10^−5^ s^−1^ is lower than that at 5 × 10^−3^ s^−1^.

## 5. Conclusions

In the present work, by conducting uniaxial tensile tests on MarBN steel at different strain rates as well as various temperatures, serrations of different morphologies were observed in the stress–strain curves. Then, the PLC effect during plastic flow was explained via the TEM photographs taken and analyzed. In summary, serrated flow under various strain rates (5 × 10^−3^ s^−1^ and 5 × 10^−5^ s^−1^) was observed in MarBN steel during tensile deformation at RT, 430 °C, and 630 °C. At a strain rate of 5 × 10^−3^ s^−1^, type-C serrations appeared under all the temperatures. The critical strain depends on the first unpinning dislocation during plastic tensile deformation due to greater vacancy concentration and dislocation density caused by the high strain rate, which decreases with the increase in the temperature until 430 °C and then increases with an increasing temperature. By contrast, type-B serrations appeared at 430 °C and 630 °C due to the low dislocation velocity caused by the strain rate of 5 × 10^−5^ s^−1^. The TEM results revealed that pinning and unpinning dislocation were responsible for the serrations at both strain rates.

## Data Availability

Data available on request from the authors.
